# Supplementary material for: Molecular Identification of the G-Protein Genes and Their Expression Profiles in Response to Nitrogen Deprivation in Brassica napus
Source: Int J Mol Sci. 2022 Jul 24;23(15):8151. doi: 10.3390/ijms23158151 (PMC9330883; doi:10.3390/ijms23158151)
Supplement: Supplementary file 1 [file ijms-23-08151-s001.zip › ijms-1802688-supplementary.pdf]

Supplementary files

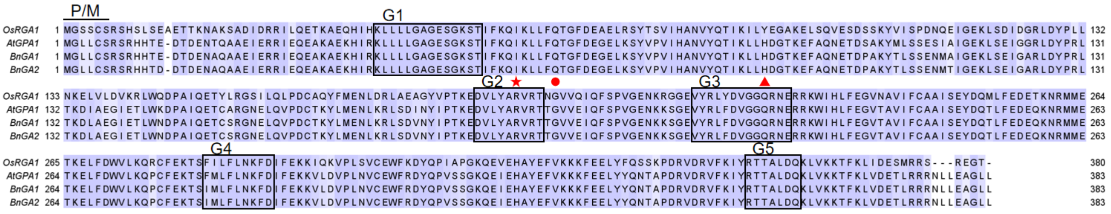

A

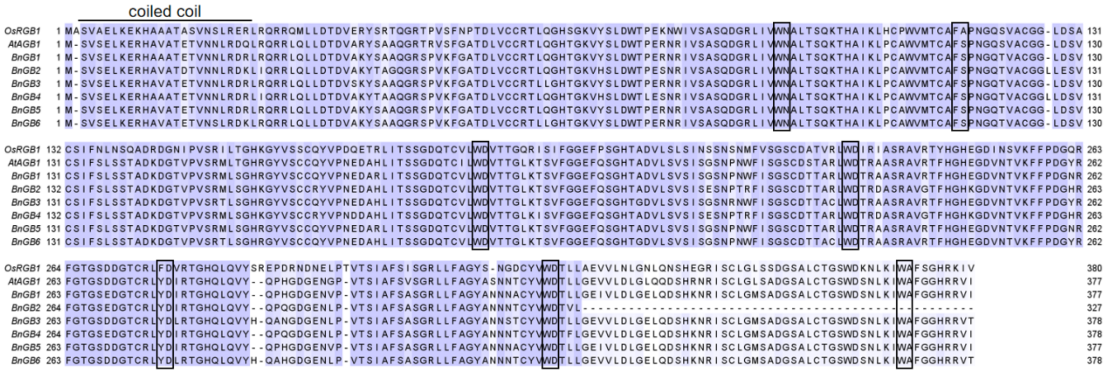

B

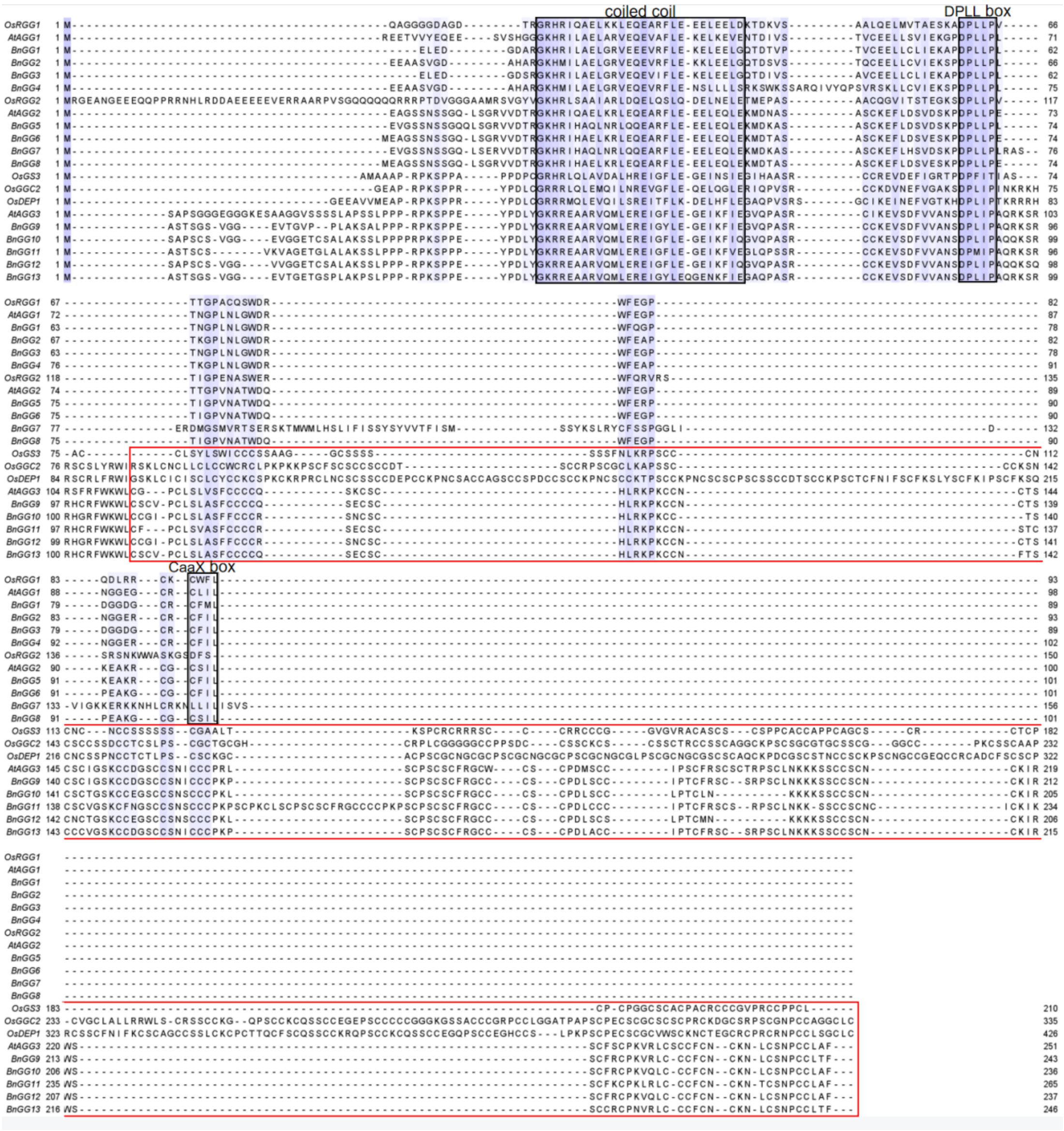

C

Figure S1. Amino acid sequence alignments of BnGα (A), BnGβ (B) and BnGγ (C) proteins from other plant species. The conserved residues are shaded in purpel. A: G1~G5 labeled the sequences for GTP binding and hydrolysis; P/M indicates the predicted sites for palmitoylation/myristoylation; ● indicates glycine (G) important for RGS interaction; ▲ indicates the conserved glutamine (Q) important for the GTPase activity of Gα proteins; ★ indicates the conserved ADP ribosylation site in Gα proteins. B: The seven tryptophan-aspartic acid (WD) repeats conserved in the Gβ proteins are marked within boxes. C: The conserved coiled-coil motif, DPLL box and the C-terminal prenylation target (CAAX) site are marked within boxes; The Cys-rich domain are marked within red boxes.

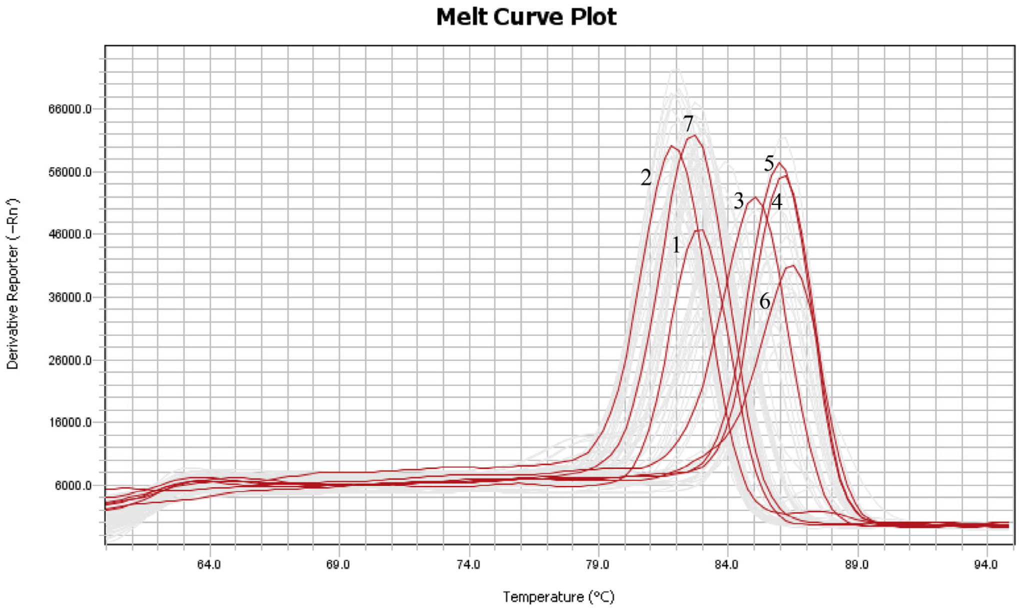

Figure S2. Melt curves of primers used for qRT-PCR.

1. q*Bnactin7*-FP/RP; 2. q*BnGA1*-FP/RP; 3. Q*BnGG9*-FP/RP; 4. Q*BnGG10*-FP/RP; 5. Q*BnGG11*-FP/RP; 6. Q*BnGG12*-FP/RP; 7. Q*BnGG13*-FP/RP.

Table S1. IDs and names of G-protein genes in plants.

group	Gene ID	Gene name
Gα	106368534	*BnGA1*
	106416165	*BnGA2*
	AT2G26300	*AtGPA1*
	Bra007761	*BraA.Gα1*
	106307271	*BolGA1*
	KJ451020	*BniB.Gα1*
	Os05g26890	*OsRGA1*
	Csa16g047880.1	*CsGα1*
	Csa07g057270.1	*CsGα2*
	Csa09g092430.1	*CsGα3*
Gβ	106346889	*BnGB1*
	106355793	*BnGB2*
	106361031	*BnGB3*
	106393802	*BnGB4*
	106434467	*BnGB5*
	111200422	*BnGB6*
	AT4G34460	*AtAGB1*
	Bra017658	*BraA.Gβ1*
	Bra034628	*BraA.Gβ2*
	Bra011536	*BraA.Gβ3*
	106295980	*BolGB1*

	106304073	*BolGB2*
	106310653	*BolGB3*
	106334918	*BolGB4*
	106337880	*BolGB5*
	KJ451022	*BniB.Gβ1*
	KJ451024	*BniB.Gβ2*
	KJ451026	*BniB.Gβ3*
	Os03g46650	*OsRGB1*
	Csa11g007470.1	*CsGβ1*
	Csa10g007670.1,	*CsGβ2*
	Csa12g009120.1	*CsGβ3*
Gγ	106415923	*BnGG1*
	106444922	*BnGG2*
	111200375	*BnGG3*
	106444926	*BnGG4*
	106378744	*BnGG5*
	106416661	*BnGG6*
	106453742	*BnGG7*
	106444151	*BnGG8*
	106370440	*BnGG9*
	106385330	*BnGG10*
	106425695	*BnGG11*
	106443216	*BnGG12*
	106409403	*BnGG13*
	AT3G63420	*AtAGG1*
	AT3G22942	*AtAGG2*
	AT5G20635	*AtAGG3*
	Bra007741	*BraA.Gγ1*
	Bra001894	*BraA.Gγ2*
	Bra023782	*BraA.Gγ3*
	Bra020117	*BraA.Gγ4*
	Bra006568	*BraA.Gγ5*
	106307696	*BolGG1*
	106341959	*BolGG2*
	106331817	*BolGG3*
	106342853	*BolGG4*
	106313974	*BolGG5*
	106325278	*BolGG6*
	106333147	*BolGG7*
	KJ451028	*BniB.Gγ1*

KJ451030	*BniB.Gγ2*
KJ451032	*BniB.Gγ3*
KJ451034	*BniB.Gγ4*
Os03g43480	*OsRGG1*
Os02g04520	*OsRGG2*
OSNPB_030407400	*OsGS3*
OSNPB_090441900	*OsDEP1*
OSNPB_080456600	*OsGGC2*
Csa16g001110.1	*CsGγ1*
Csa07g001190.1	*CsGγ2*
Csa05g095780.1	*CsGγ3*
Csa15g041710.1	*CsGγ4*
Csa19g033230.1	*CsGγ5*
Csa01g027310.1	*CsGγ6*
Csa13g023540.1	*CsGγ7*
Csa08g014440.1	*CsGγ8*
Csa20g032610.1	*CsGγ9*

Table S2. Segmentally duplicated genes in *B.napus.*

**segmentally duplicated genes**	
BnGB3	*BnGB5*
BnGB6	*BnGB4*
BnGB6	*BnGB3*
BnGB6	*BnGB5*
BnGG4	*BnGG1*
BnGG9	*BnGG13*
BnGG12	*BnGG9*
BnGG12	*BnGG13*

Table S3. Primers used for subcellular localization of G-protein genes.

Gene name	Gene ID	Sequence of forward primer (5′-3′)	Sequence of reverse primer (5′-3′)
BnGG10	106385330	actcttgaccatggtagatctGGATGTCTGC TCCTTCATGCA	atcctaggactagtcagatctGAAAGCTAAAC AACAAGGATTAGAACA
BnGG11	106425695	actcttgaccatggtagatctGGATGTCTGC TCCTTCATGCA	actcttgaccatggtagatctATGGCTTCTACT TCATGCAGTGTC
BnGG12	106443216	actcttgaccatggtagatctGGATGTCTGC TCCTTCATGCA	actcttgaccatggtagatctATGTCTGCTCCT TCATGCAGTG

Table S4. The expression values of G-protein in 12 *Brassica napus* tissues across different developmental stages.

Gene ID	flower	leaf	slique	stem	sepal	pistil	stamen	ovule	pericarp	blossomy pistil	wilting pistil	root
BnGA1	0.00	0.00	0.00	0.00	0.00	0.00	0.00	0.00	0.00	0.00	0.00	0.00
BnGA2	0.00	0.00	0.00	0.00	0.00	0.00	0.00	0.00	0.00	0.00	0.00	0.00
BnGB1	37.97	30.47	47.58	57.08	14.79	103.47	2.90	64.57	23.40	9.07	2.51	28.80
BnGB2	12.97	24.66	18.85	41.11	34.02	27.89	4.92	27.74	28.50	18.79	24.55	52.06
BnGB3	1.92	0.32	2.49	1.08	1.86	8.38	0.15	0.74	4.73	2.41	2.99	21.55
BnGB4	0.77	0.24	0.39	0.25	0.13	0.15	0.00	0.00	2.20	0.00	0.16	0.96
BnGB5	5.07	3.04	6.21	4.57	6.90	9.05	1.03	3.63	6.06	15.16	30.14	32.84
BnGB6	22.52	50.15	30.00	49.43	50.45	27.44	6.54	23.89	42.71	36.99	51.21	95.96
BnGG1	39.44	13.13	35.77	16.76	18.20	32.75	7.61	27.26	36.80	3.84	4.76	18.99
BnGG2	0.00	0.00	0.00	0.00	0.00	0.00	0.00	0.00	0.00	0.00	0.00	0.00
BnGG3	61.80	21.78	42.67	42.09	27.44	32.44	8.70	69.49	36.14	16.71	17.36	37.06
BnGG4	0.00	0.00	0.00	0.00	0.00	0.00	0.00	0.00	0.00	0.00	0.00	0.00
BnGG5	28.47	49.43	31.40	45.95	43.39	22.79	9.36	10.31	23.11	28.53	32.73	24.40
BnGG6	7.53	7.19	9.45	18.25	11.27	8.61	3.84	10.57	13.46	17.41	17.12	23.10
BnGG7	0.00	0.00	0.00	0.00	0.00	0.00	0.00	0.00	0.00	0.00	0.00	0.00
BnGG8	14.91	14.23	15.22	30.84	15.82	6.95	3.84	13.21	3.80	13.30	19.82	17.73
BnGG9	0.00	0.00	0.00	0.00	0.00	0.00	0.00	0.00	0.00	0.00	0.00	0.00
BnGG10	7.39	0.00	0.21	0.00	0.00	1.67	0.10	3.64	0.25	0.00	0.00	0.00
BnGG11	0.00	0.00	0.00	0.00	0.00	0.00	0.00	0.00	0.00	0.00	0.00	0.00
BnGG12	9.49	0.00	0.34	0.00	0.00	2.98	0.00	8.83	0.00	0.00	0.00	0.14
BnGG13	0.00	0.00	0.00	0.00	0.00	0.00	0.00	0.00	0.00	0.00	0.00	0.00

Table S5. Primers used for RT-qPCR analysis of G-protein genes.

Gene name	Gene ID	Sequence of forward primer (5′-3′)	Sequence of reverse primer (5′-3′)
BnGA1	106368534	AGATCTACAGGACGACGGCT	AAAAGGCCAGCCTCCAAGAG
BnGG9	106370440	TGGAAGTGGCTCTGTAGCTG	GCAGCACTTGGGCTTCCTTA
BnGG10	106385330	TGCACAACAAAAGAGTCGAAGG	CAGCTGCAACTTGTACAGCA
BnGG11	106425695	GAAGTGGCTCTGTTTCCCGT	AGCATTTGGAACCAACGCAG
BnGG12	106443216	CTAGCTAAGTCGTCCCTGCC	CTAGCATCTGGACTCTCGCC
BnGG13	106409403	TGGAAGTGGCTCTGTAGCTG	GACCCGTCACAGCATTTGGA

All qRT-PCR primer sequences for G-protein genes were obtained from the NCBI primer-blast (https://www.ncbi.nlm.nih.gov/tools/primer-blast/).
